# A Qualitative Analysis of Emergency Medical Services (EMS) Personnel Experiences and Perceptions Responding to Drug Overdoses in the United States (US) During the COVID-19 Pandemic

**DOI:** 10.56808/2586-940x.1045

**Published:** 2023-01-20

**Authors:** Nae Y. Won, Joseph J. Palamar, Stephen A. Mike, Nicole D. Fitzgerald, Linda B. Cottler

**Affiliations:** a Department of Epidemiology, College of Public Health and Health Professions, College of Medicine, University of Florida, USA; b New York University Grossman School of Medicine, Department of Population Health, USA

**Keywords:** Emergency medical services, Substance use, Opioids, Overdose, COVID-19, Prehospital care

## Abstract

**Summary:**

**Background:**

The purpose of this work was to describe the experiences of EMS personnel in responding to drug overdose-related calls and the impact the pandemic has had to help better inform current response and treatment efforts.

**Methods:**

Semi-structured interviews were conducted with 99 EMS personnel across 18 areas throughout the United States that were designated as Early Warning Network sentinel sites by the National Institute on Drug Abuse-funded National Drug Early Warning System. Participants were asked about topics including the potential burdens from the pandemic and the opioid crisis. We coded the interview responses and identified themes through qualitative analysis. Multiple cycles of descriptive coding, recoding, subcoding, pattern-coding, and thematic coding of responses were conducted.

**Results:**

Responses were categorized into the following themes: 1) being over-worked from increased call volume; 2) increased risk for personal harm when responding to patients; 3) compassion fatigue due to long hours and repeat calls for the same people; 4) conflicting perceptions of the utility of naloxone; 5) the need for better treatment options to respond to opioid crisis on top of COVID-19.

**Conclusions:**

The burden of the substance use disorder (SUD) crisis on EMS personnel has been compounded by the COVID-19 pandemic. These reports from EMS personnel throughout the US can help inform policy and procedures to better protect the mental health of EMS personnel and to ensure better care for patients with SUD. These experiences and recommendations may be of use for other countries as substance use and COVID-19 are global health issues.

## 1. Introduction

Emergency medical service (EMS) personnel are frontline health care workers, and as such, they are more likely to encounter both fatal and non-fatal overdoses [1]. EMS personnel have been used as a primary data source for surveillance of non-fatal opioid overdose through their naloxone hydrochloride (also commonly known by the brand name Narcan, which henceforth will be referred to as simply “naloxone”) administration data [2]. Naloxone is an opioid antagonist, used to rapidly reverse opioid drug overdoses. Recent research shows that EMS naloxone administration increased by 75% from 2012 to 2016 [2], suggesting an increase in overdose response burden among EMS personnel. Not only do these personnel treat drug overdoses, but they can provide harm reduction and outreach to patients through recommendations for treatment and community outreach, including peer support services [3].

Since spring 2020, the severity of the opioid crisis in the US has been compounded by the COVID-19 pandemic. On March 11, 2020, the World Health Organization declared COVID-19 a pandemic. From June 2020 to June 2021, the Centers for Disease Control and Prevention (CDC) reported 101,263 overdose deaths in the US, which is a 20.6% increase from the previous year [4,5]. Increases may have been due, in part, to stay-at-home orders and social distancing measures implemented to combat the pandemic [6,7]. Social isolation often led to increased anxiety and depression levels among individuals as well as reduced social and recovery support, and health care services for individuals with opioid use disorders [6,7]. Reduced resources and social isolation also contributed to a lower number of people in proximity to help reverse an overdose [7].

EMS personnel offer a unique perspective on the current substance use crisis in real-time, including the impact of the COVID-19 pandemic [8]. Several studies reported the prevalence of EMS dispatches for overdose events during the COVID-19 pandemic [9–12]. Nationwide, EMS activation increased almost two-fold [10]. Individual state EMS data also suggest an increase in opioid-related EMS visits, as well as naloxone administrations [11]. Even before the pandemic, studies began to demonstrate burnout among EMS personnel due to rigorous work demands and low resources with respect to responding to opioid overdose-related emergencies [13–15]. The burnout among EMS personnel may have then been compounded with their vital role in both the COVID-19 pandemic and the opioid crisis. This study aimed to acquire the unique perspectives of EMS personnel regarding their current responses to overdose-related calls and the perceived impacts that COVID-19 has had on drug-related mortality and emergency responses.

## 2. Methods

### 2.1. Study design and participants

This study was conducted as a component to our National Institute on Drug Abuse (NIDA)-funded National Drug Early Warning System (NDEWS) project [16,17]. Semi-structured telephone interviews were conducted with EMS personnel across 18 cities and states throughout the US designated as Early Warning Network sentinel sites by NDEWS. The 18 sites included some specific cities, metropolitan areas, and entire states: Atlanta Metropolitan Area, Georgia; Chicago, Illinois; Denver, Colorado; Detroit, Michigan; Southeastern Florida; Los Angeles, California; state of Maine; Minneapolis, Minnesota; New York City, New York; Philadelphia, Pennsylvania; Phoenix, Arizona; San Diego, California; San Francisco, California; Seattle, Washington; St. Louis, Missouri; state of Texas; Washington, District of Columbia (DC); and state of West Virginia. R 3.5 software [18] was used to randomly select EMS agencies for recruitment based on publicly listed contact information online. EMS personnel were identified by their title when trained interviewers contacted their agencies via telephone. The trained interviewer described the study and assessed eligibility criteria, which included: 1) being 18 years or older, 2) working as an EMS chief, director, staff, or paramedic, 3) having worked in their specific role/location for at least six months, and 4) currently working in the specified role. All interviewers were screened by the study team, received interviewing and study protocol training, in addition to Institutional Review Board (IRB) Human Subjects Research and Health Insurance Portability and Accountability Act (HIPAA) trainings. All interviewers were from the University of Florida. EMS chiefs and directors were included if they had first-hand experience in responding to occasional calls. They also provided insight into the experience of multiple paramedics and staff given their role or reviewing reports provided by those on the field. Interviews were conducted from October 2020 to August 2021. We enrolled 110 eligible participants in the study, who provided informed consent. Eligible participants were interviewed at baseline and asked to retrospectively compare events in the past 30 days to past events overall, such as months in which COVID-19 cases were perceived to be the highest. Eleven out of 110 were excluded from the analysis due to unavailable transcripts or poor quality of the interview recording. We conducted follow-up interviews at 30-day intervals over a period of five months.

The average duration of each interview was 30 min. Most of the survey was quantitative in nature, but seven questions on the baseline and six questions on the follow-up survey were open-ended to obtain richer qualitative responses (the focus of this analysis). Qualitative responses were coded from the following open-ended question topics: 1) the burden to EMS personnel as a result of the ongoing drug overdose crisis, 2) the burden to EMS personnel as a result of the COVID-19 pandemic, 3) whether they wanted to share anything as a first responder with the director of NIDA, 4) whether there were specific overdose cases that affected them, 5) thoughts on the role of naloxone in reversing an overdose, and 6) anything they felt they should share regarding drug use or overdoses in their area. The additional question on the baseline survey was the type of drugs involved in the overdose calls.

Participants received a $10 gift card at baseline and a $5 gift card for each of the four follow-up interviews to compensate them for their time. They received an additional $20 gift card for completing all five interviews for a possible total of $50 as an incentive for participating in this study.

### 2.2. Ethical issues

The Institutional Review Board at the University of Florida (IRB#: 202000579, 2/25/2022) reviewed and approved all study protocols.

### 2.3. Data analysis

Multiple cycles of descriptive coding, recoding, subcoding, and pattern-coding of responses were conducted [19] using Atlas.ti version 8 software [20]. Thematic coding was conducted in an inductive manner with some definition changes and code-splitting [21,22]. A second team member double-coded 10% of interviewer notes for quality control. Dominant and/or repeated codes were then categorized into themes. Quotations that adequately summarized specific topics were summarized along with themes in order to present a comprehensive picture.

## 3. Results

### 3.1. Themes

A total of 99 participants were included for this qualitative analysis (Table 1).

We identified the following themes: 1) being over-worked from increased call volume; 2) increased risk for personal harm when responding to patients; 3) compassion fatigue due to long hours and repeat calls for the same people; 4) conflicting perception on the utility of naloxone; and 5) the need for better treatment options to respond to overdose and COVID-19. The representative quotes for each theme can be found in Table 2.

#### 3.1.1. Being over-worked from increased call volume

All participants (n = 99, 100%) mentioned that call volume overall increased during the pandemic. A few participants (n = 2, 2%) working in less populated areas noted a minimal increase. Call volume increased not only due to COVID-19 related calls, but also due to increased suicide attempts and overdoses. Further, adding to this burden, calls for minor conditions or “unnecessary reasons” such as the sniffles increased. Overdoses reportedly increased in most districts during COVID-19. Participants also noticed a higher prevalence of overdose among adolescents and patients from varying socio-demographic backgrounds compared to times prior to COVID-19. Spikes and clusters of overdoses also reportedly occurred during the pandemic when stimulus checks arrived to the public or when a particularly “bad batch” of drugs (e.g., containing fentanyl) reached the local community. Given that EMS is the only medical service utilized by people who use drugs in some of these areas, increases in drug-related emergencies placed great strain on the full EMS system and led to reported burnout among EMS personnel.

The increase in EMS call volume often led to seemingly unending workdays. Some participants (n = 5, 5%) reported having to work longer hours, double shifts, and/or more days per week, even when they were supposed to be under quarantine. This was associated with greater concern that EMS personnel would contract COVID-19 and infect their families at home. Further, the extra hours worked tended to take participants away from home, reduce personal time and vacation, and add to the challenges of finding childcare while they worked long shifts. Some participants mentioned lack of overtime pay; some participants who reported being over-worked were volunteers. Strain related to the extra work reportedly led to high turnover rates with some EMS personnel quitting to search for less stressful jobs. Those who remained often entrusted larger caseloads. In addition, there were comments about EMS staff feeling underappreciated and often being viewed as “ambulance drivers”, which contributed to the burnout.

#### 3.1.2. Increased risk for personal harm when responding to patients

Most participants (n = 91, 91.9%) mentioned an increase in risk of personal harm when responding to patients due to the risk of COVID-19 infection and/or combative patients. At the beginning of the pandemic, procedures were implemented, such as equipping personal protective equipment (PPE) for all contact with patients to reduce the risk of infection. PPE was commonly cited as a major source of stress. PPE typically consisted of N-95 masks, face shields, gloves, and occasionally gowns. All patient visits commonly needed PPE, as EMS personnel often had to assume patients were infected with COVID-19. Having to wear an N-95 mask for all visits was frequently reported as a stressor. Wearing these masks for an extended period can lead to difficulty in breathing and foggy glasses or goggles. Some also experienced having allergic reactions to extensive use of gear. During summer months, PPE often led to overheating. Some reported difficulty communicating and connecting with patients or among staff while wearing masks and face shields. In response to the recurrent challenges, some participants reported “COVID fatigue,” in which they became careless regarding use of PPE during visits.

EMS personnel mentioned devoting a large portion of time to the process of “suiting up” and “suiting down” with PPE before and after each visit throughout the day. EMS personnel also had to engage in extensive disinfecting practices after each visit (e.g., disinfecting, handwashing, showering, changing of clothes, disinfecting the ambulance). All of these procedures reportedly led to slower response times with many visits taking much longer than usual. As such, while PPE helped protect EMS personnel and patients alike, use inevitably led to delays in emergency response, a critical factor for an overdose as well as other condition.

In addition to discomfort related to PPE, PPE conservation was critical throughout the pandemic. Early in the pandemic, in particular, many departments experienced PPE shortages. Some participants mentioned that in order to save PPE, only one EMS personnel would enter the premises to respond to a call. Participants described this situation as leading EMS personnel to enter potentially dangerous situations alone. Some environments are particularly dangerous, unstable, or uncontrolled in general. Thus, EMS personnel entering such an environment alone had potentially increased risk of experiencing violence. Four of the participants noted that a patient became combative while addressing the call. Others reported patients in psychotic states, some of which had weapons nearby. This danger was in addition to the usual risk of needle sticks.

#### 3.1.3. Compassion fatigue due to long hours and repeat calls

Most participants observed other EMS personnel lacking compassion towards overdose patients or even patients in general because they were over-worked (n = 93, 93.9%). Many reported “compassion fatigue” resulting not only from large caseloads, but also from aiding the same overdose patients “day in and day out.”

EMS personnel are often called to respond to the same patient, sometimes more than once in the same 24-h period. At times, these patients are referred to as “frequent fliers” due to perceived constant need of assistance, which only added to the stress of handling COVID-19 cases. Some participants further complained that having to assist the same overdose patient multiple times took away attention and resources from other emergencies, such as a person having a stroke or heart attack. Others noted that repeated visits “feels like a waste of resources” or that “overdose is irresponsible and self-induced” as opposed to more “legitimate” illnesses such as contracting a virus. Adding to their frustration, some participants felt that people addicted to opioids sometimes had a feeling of complacency with their condition, and do not desire help or change. Some EMS personnel experienced some patients were not appreciative of being revived. Other patients were reportedly uncooperative or angry, and some patients were said to have even physically assault EMS personnel. Dishonesty from patients and parents of adolescent patients was also another concern participant raised as misinformation can complicate treatment. A few participants reported that due to chronic exposure to this situation, their morale was destroyed, and it skewed their views about society overall. A few participants stated that this situation is not what EMS personnel sign up for as part of the job.

Even with the frustration mentioned above, witnessing a patient die appeared to weigh on the EMS personnel. In smaller communities, EMS personnel often personally knew patients who overdosed. Others had a particular “soft spot” for people addicted to opioids, especially if they personally knew someone with an addiction or someone who died of an overdose. Some participants shared onerous personal stories, such as having to revive parents who overdosed in front of their children and having to tell family members that their child died from an overdose. Despite the exhaustion EMS personnel experienced from the increased overdose and COVID-19 9–1–1 calls, they continued to reiterate the importance of not disregarding the opioid epidemic.

#### 3.1.4. Conflicting perceptions on the utility of naloxone

Of all the study participants, 78% mentioned their views about naloxone. While most (n = 53, 53.5%) participants supported naloxone use, almost a third (n = 24, 24.2%) were in aversion or ambiguous towards the utility of naloxone. Some participants stated it enabled people to use drugs and enter a cycle of overdose and reversal; thus, naloxone was creating a cycle merely kept them alive rather than treating their condition. Naloxone was sometimes described as a Band-Aid for the drug problem. One EMS personnel said it is “empowering these people to keep overdosing.” Some EMS personnel mentioned that patients tend to immediately leave the scene after a naloxone overdose reversal without further medical care. Additionally, some EMS personnel witnessed patients who are brought to the emergency department (ED) leave against medical advice, which led them to believe that many individuals have little to no desire to receive help or treatment. Some participants described naloxone as problematic because some patients who use opioids consider naloxone reversal as a status symbol or a “badge of honor” translating to being a “more extreme user.” For example, one participant described a patient he just saved asking in awe how many shots it took to reverse his overdose “this time,” also known as naloxone reversal competitions. Some participants in favor of naloxone (n = 15, 15.1%) complained about lack of access to naloxone, and said they did not feel properly equipped to handle overdoses without it. This has become even more critical as more naloxone is often necessary with the increased need for opioid overdose reversal. EMS personnel from smaller departments in particular stated the lack of adequate access to naloxone as a concern. Other participants implied they did not know where to obtain naloxone. Participants also mentioned funding as another impediment to naloxone access, such as increased cost of naloxone, issues of reimbursement, and financial dependence on local politics.

There were also discordant views regarding naloxone use by the community. While some participants promoted use of naloxone by the public and even left doses behind for patients, others complained about naloxone access by nonmedical personnel. Participants commented on the perceived problematic nature to distribute it over the counter, and that naloxone use should be limited to medical personnel. Some said medically unsupervised home-reversal could lead to underreporting of overdoses; consequently, it may prevent EMS providing treatment and preventative education to patients with an overdose. Participants also mentioned that some patients became combative or violent after a reversal, which could place others who administer the naloxone in danger. Another concern was the potential for delay in calling 9–1–1 in an overdose event. Specifically, if a medically unsupervised home-administered dose of naloxone does not work, then it may be too late for reversal via EMS. One participant described these contrasting perspectives as a “political division” among EMS personnel.

#### 3.1.5. Need for better treatment options to respond to overdose and COVID-19

All participants (n = 99, 100%) emphasized that more treatment options are needed for patients they encounter with an overdose. As one participant stated, people who overdose need to be able to “get help beyond today.” Our qualitative findings suggested that effective services were already difficult to acquire. But access reportedly became even more limited after the onset of the COVID-19 pandemic because discerningly most attention was dedicated to COVID-19 patients.

While many participants stressed the need for services and treatment beyond EMS (and typical naloxone treatment), it was commonly reported that EMS personnel still need more funding and staff to help handle the current need. Some mentioned the need for collaboration with other organizations (e.g., local police). One participant mentioned that a mental health professional should be available to respond to certain patients with mental health needs when addressing an overdose call. Currently, most EMS personnel do not have options other than to take overdose patients to a local ED. A few participants recommended being able to engage in more community outreach and education about treatment options and referrals because EMS is often the only medical care some patients receive. Participants indicated that particularly during the COVID-19 pandemic and hospital overcrowding, access to EDs and hospitals, along with acute and chronic treatment options, was limited for patients with an overdose.

Services were, described by participants, as lacking even before the pandemic; but the situation exacerbated when even fewer resources became available. Testing positive for COVID-19 or not having been tested due to lack of test kits available were other potential barriers to treatment at the beginning of the pandemic. Mental health and psychiatric services in particular were regarded as lacking; and this dearth of services was particularly harmful during the pandemic due to increases in need for those with substance use disorder perhaps due to social isolation, especially in rural areas and underserved communities. Participants further accentuated the need for innovative treatments focusing on repeat overdose patients. These treatment options should assist with formulating a plan with the patient, follow-up with the patient, and retain the patient throughout the entire process. This highlights the need for more social workers and mental health professionals. EMS participants recommended other programs, such as telemedicine, hotlines, and detoxification programs as alternative treatment options; however, they emphasized personal contact as a necessary component of treatment.

## 4. Discussion

This qualitative study provided insights into the experiences of EMS personnel in responding to substance use-related calls, especially during the COVID-19 pandemic on top of the opioid crisis in the US. EMS personnel are critical stakeholders in combating the opioid crisis; they are often the first health care provider called to the overdose scene [1,23]. Therefore, it is essential to understand the needs of EMS personnel directly from them to provide the resources and training needed to address those needs, which our findings provide. The main themes in this study were: 1) being over-worked from increased call volume, 2) increased risk for personal harm when responding to patients, 3) compassion fatigue due to long hours and repeat calls for the same person, 4) conflicting perceptions of the utility of naloxone, and 5) the need for better treatment options to respond to overdose and COVID-19.

Two themes all of our participants mentioned were increased call volume and the need for more and better treatment options. The literature confirmed that EMS personnel experienced an increase in overdose calls during the pandemic with high burnout rates due to poor sleeping patterns, irregular eating habits, and emotional exhaustion [10,11,24–27]. Our study found that the increases in call volumes, long work hours, fear of COVID-19 contamination, physical and emotional exhaustion, and feeling under-appreciated contributed to this high burnout rate. Another contributing stressor for EMS staff during the pandemic was the PPE mandate because of its discomfort and hindrance on the performance [28,29].

Despite the ongoing opioid crisis, EMS participants observed limited and reduced treatment options for substance use disorders (SUDs) during the pandemic. As Shreffler et al. found, substance use treatment facilities reduced capacity to enable social distancing to mitigate COVID-19 transmission [30]. Decreases in the availability of resources, support systems, and medication-assisted treatment led to higher relapse and overdoses [30]. Responders also reported difficulty referring patients to outpatient opioid treatment programs during the pandemic due to reduced services [31]. Furthermore, the pandemic shifted how SUD treatment was provided, reducing the personal contact that our study participants identified to be a significant part in the treatment process. Results from a longitudinal study of 15,691 outpatient behavioral health treatment facilities in the US by Cantor et al. suggested a shift to more telehealth options—a 77% and 143% increase from 2020 to 2021 for mental health treatment and SUD treatment facilities, respectively [32]. A limitation of these telehealth services was access to computers, Internet, and health insurance [32,33]. Our study corroborated these findings along with several EMS personnel desiring long-term solutions for the opioid crisis and more opportunities for collaboration with other stakeholders to address the opioid crisis.

Another notable finding was the mixed perception of EMS on the utility of naloxone. Though many EMS personnel stressed that naloxone is effective and critical in reducing harm, others expressed dissatisfaction with naloxone as a solution to the opioid crisis. One common concern among those dissatisfied was that the public availability of naloxone could decrease EMS encounters with overdose patients, which would reduce the potential opportunities to educate and connect patients to treatment and resources after an overdose reversal. However, community naloxone distribution can also be an opportunity for people who use drugs to be connected with resources through various stakeholders involved in supporting those with SUD, such as community health organizations and health care providers [28,29]. It is important to address naloxone misperceptions among EMS personnel who work in remote areas, given the literature on the effectiveness of naloxone in reducing overdose deaths [34–40]. Supplemental to training, collaborative efforts with public health professionals may help address EMS personnel concerns regarding the opioid crisis and the use of naloxone and reduce the burdens experienced by these individuals.

## 5. Conclusions

EMS personnel experienced burnout due to increased work volume and responding to repeat patients. Further, many EMS personnel appeared to struggle with treatment options for overdose patients with SUD, as fewer options for treatment are available during the pandemic. We believe these reports can help inform policy and procedure to better protect the mental health of EMS personnel and ensure better care for patients with SUD globally. Substance use is a rising global health concern as more mental health issues are exacerbated during the COVID-19 pandemic. It is important globally to understand possible burdens for EMS personnel and recommended training that may help EMS personnel.

## 6. Recommendations

### 6.1. Limitations

Despite this study being a multi-center study throughout the US, participants may not be representative of all EMS personnel in the US. Given that EMS personnel have generally been over-worked during the pandemic, it is unknown whether this affected study participation and whether this affected responses. Some EMS personnel might have felt too uncomfortable disclosing some information, while others might have simply used the study as an opportunity to report work stressors. As such, it is unknown to what extent situations and emotions reported are representative of an EMS personnel’s typical workday.

### 6.2. Implications

However, this study provides a better understanding of EMS personnel needs, especially in providing care for substance overdose during public health crisis as the COVID-19 pandemic. As highlighted previously, EMS personnel often are the first health care providers called to the overdose scene globally; therefore, it is pivotal to address their needs to enhance future care. Though this study focuses on the US, substance use and the COVID-19 pandemic are international public health concerns. Hence, our findings may provide better insight to alleviate possible burdens EMS personnel face in care delivery for drug-related overdoses worldwide.

## Figures and Tables

**Table 1.** Participant characteristics from study of Emergency Medical Service (EMS) personnel (N = 99).

Characteristics	N	Percent
Age (Years) 18–24	2	2.0
25–34	8	8.1
35–44	19	19.2
45–54	42	42.4
55–64	21	21.2
65+ Site Location (City, State or Name of State)	7	7.1
Atlanta, Georgia	9	9.1
Chicago, Illinois	7	7.1
Denver, Colorado	5	5.1
Detroit, Michigan	6	6.1
Southeastern Florida	5	5.1
Los Angeles, California	7	7.1
State of Maine	7	7.1
Minneapolis, Minnesota	5	5.1
New York City, New York	4	4.0
Philadelphia, Pennsylvania	4	4.0
San Francisco, California	2	2.0
Seattle, Washington	2	2.0
St. Louis, Missouri	7	7.1
State of Texas	23	23.2
Washington, DC	4	4.0
State of West Virginia	2	2.0
Job Title EMS Chief	69	69.7
Paramedic	12	12.2
Emergency Medical Technician (EMT)	10	10.2
Other	8	8.1

**Table 2.** Quotes from emergency medical service (EMS) qualitative responses representative of the coded themes (n = 99).

Theme 1: Being over-worked from increased call volume n = 99 (100%)

• EMS can’t work short [staffed] and need a [minimum] number of staff all the time. We had to ask people to do overtime when [other] people couldn’t work. We had burnout from extra steps for prevention. The overtime work on the remaining staff was challenging and was one of the biggest negatives that affected us during the pandemic. (Texas)
• We dealt with the [drug abuse] crisis prior to COVID, with people being cooped at home and not being able to get out [during COVID] exacerbated the crisis. We not only have COVID thrown at us, but we also saw an increase in overdoses. We have seen a much larger increase in cocaine/fentanyl use; it is noticeable in the streets. (New York)

**Theme 2: Increased risk for personal harm when responding to patients n = 91 (91.9%)**

• You have to gown up now, which takes time and effort. Putting a mask on a patient is new and we’ve never had to do that before. [We] have to stock COVID supplies and make sure you have enough. More logistics mean slower response times for patients. (California)
• Drug abuse calls are very dangerous [for EMS personnel]. People [who overdose] tend to be in a psychotic state, [and they] may have weapons at their disposal. Individuals may become irate and violent, [and the patient] might have to be subdued. One time we found a loaded revolver [on the scene]. Hypodermic needles [also found at the site] might be infectious. If someone [patient] is yelling or acting in an odd manner, it could be a psychological strain [on EMS staff]. (California)
• We required people to put on PPE regardless of call type; it restricts movements and breathing, especially in the summer where the heat and humidity cause discomfort. (Minnesota)
• PPE is incredibly uncomfortable, and we have to wear it for every call because most of those calls are of shortness of breath [symptom for COVID-19]. PPE fatigue and COVID fatigue are felt. General fatigue has definitely hit us pretty hard. (Washington DC)

**Theme 3: Compassion fatigue due to long hours and repeat calls for the same person n **=** 93 (93.9%)**

• The burden is that all the attention has gone to this illness [COVID-19], including money, resources, and equipment. This overshadowed the current opioid crisis. People began forgetting about the issue [opioid crisis] and how this is an illness that needs to be treated. (Texas)
• We keep seeing the same people over and over again, they are not able to get the help they need. (Missouri)
• [I want to] make sure that we don’t forget one epidemic because we have a pandemic. The opioid epidemic doesn’t go away because of COVID. Don’t push one epidemic to the back burner; keep the opioid epidemic in the public eye and have resources. The opioid epidemic is actually worse now. (West Virginia)
• The greatest burden is responding to multiple calls for the same person over and over. We developed a quick response team [for substance use], which visits people after overdose events, and they link them to treatment and our first responders have bought into that and it’s given them hope. EMS personnel want to fix things and they are not able to do that. (West Virginia)

**Theme 4: Conflicting perceptions of the utility of naloxone n = 77 (77.8%)**

• Naloxone used to cost $36 for six doses and now it’s over $400. (Texas)
• Sometimes we have trouble obtaining naloxone from the suppliers. The cost [of naloxone] has gone up 200 times. In previous years, [naloxone pre-filled syringes] were 6 or 8 dollars, and now they are $45.90 for pre-filled naloxone. Another thing is, now we have to give 4–6 mg [of naloxone] when we use to give 1–2 mg because they weren’t consuming such combination of drugs [previously]. (Texas)
• Naloxone only seems to allow them [patients] to use again tomorrow. We need to stop them [from using]. Most efforts in EMS are to keep people alive, not to stop using. (Texas)
• Having naloxone regularly available to addicts might keep them alive, but it allows them to continue abusing drugs over and over. (Texas)
• I’m not 100% sure of getting naloxone over the counter. You’re not helping them get off drugs; you’re just prolonging it and letting them do it over and over again. Many of the government-run programs allow them to keep going [with drug use]. We give naloxone to the same people on a weekly basis. I don’t think the current programs that exist are solving the problem; they’re just keeping them alive. It’s a cycle. (Texas)
• [These] two guys wanted to know how much naloxone it took to bring them [back] around. It’s disheartening to know that they do this for pleasure or as a competition. (New York)

**Theme 5: The need for better treatment options to respond to overdoses and COVID-19 n **=** 99 (100%)**

• Just because there’s a pandemic going on doesn’t mean drug abuse and drug abuse services are any less important. (Texas)
• Even before COVID, there were no places to take them-inpatient or outpatient services. [What we find is] 95% [of the patients] have mental health issues on top of drug abuse and drug addiction, and there is no place to take them. (Missouri)
• The biggest burden [is] we can treat [people] for acute drug overdoses, but [there are only] a few services [in the community that can] help in the long-term. (Georgia)
• We need to find a way to work directly with the hospital so that when you have a person who overdosed in the hospital you don’t just let them walk out of there without [a plan for] treatment. (Georgia)
• First responders need more education and resources about how to treat [drug overdose] long-term, not just [with] episodic” treatment. (Maine)
• Send us resources. Provide resources in the community—especially in rural places—so that people who have SUD [substance use disorder] have places to go besides the ER. Putting people on suboxone in the ER and letting them out becomes a revolving door. If you’re involved in addiction care, you know this is not a solo act. These resources might be necessary for years. Fund treatments in every corner. (Maine)
• Personal interaction is very important. We have taken out the interaction [because of COVID]; it really affected the individuals that we are calling. [Some patients] don’t have access to a phone or a computer to do virtual visits. Personal contact is really important. (West Virginia)
